# Eye-closure increases children's memory accuracy for visual material

**DOI:** 10.3389/fpsyg.2014.00241

**Published:** 2014-03-24

**Authors:** Serena Mastroberardino, Annelies Vredeveldt

**Affiliations:** ^1^Neuroimaging Laboratory, Santa Lucia Foundation, IRCCSRome, Italy; ^2^Department of Criminal Law and Criminology, Faculty of Law, VU University AmsterdamAmsterdam, Netherlands

**Keywords:** children, eye-closure, memory retrieval, investigative interviewing, cognitive load, modality-specific interference

## Abstract

Research shows that closing the eyes during retrieval can help both adults and children to remember more about witnessed events. In this study, we investigated whether the eye-closure effect in children is explained by general cognitive load, modality-specific interference, or a combination. 120 children (60 female) aged between 8 and 11 years viewed a 5-min clip depicting a theft and were questioned about the event. During the cued-recall interview, children either viewed a blank screen (blank-screen condition), kept their eyes closed (eye-closure condition), were exposed to visual stimuli (visual-distraction condition), or were exposed to auditory stimuli (auditory-distraction condition). Children in the blank-screen and eye-closure conditions provided significantly more correct and fewer incorrect responses about visual details than children in the visual- and auditory-distraction conditions. No advantage was found for auditory details. These results support neither a pure cognitive-load explanation (in which the effect is expected to be observed for recall of both visual and auditory details), nor a pure modality-specific account (in which recall of visual details should only be disrupted by visual distractions). Practical implications of the findings are discussed.

## Introduction

One critical point in criminal investigation, especially in the early stages, is gathering evidence through questioning the witness/victim. When interviewing a child, this stage becomes even more crucial due to the cognitive and psychological factors affecting performance of this particular group of witnesses. In fact, children tend to report less information compared to adults, despite being generally accurate (see Goodman and Melinder, 2007, for a review), and may experience difficulties in focusing their attention for prolonged times. They are also prone to be influenced by situational factors such as the characteristics of the interviewer (e.g., age and status) and of the interview itself (e.g., social cues and types of requests; see Krähenbühl and Blades, 2006; Quas et al., 2007). In order to overcome these issues, researchers have developed or adapted a number of interview protocols with the purpose to help professionals gather accurate information from a child witness (e.g., Cognitive Interview: Fisher and Geiselman, 1992; Stepwise Interview: Yuille et al., 1993). These protocols generally help the child to remember more accurately, but there is also evidence of a small increase on the number of errors (see, for example, Memon et al., 1997 for a meta analysis on the Cognitive Interview).

In addition, although experts in many countries are trained in one or more of such protocols, surveys with police officers and other professionals, such as social workers, show that in many cases they incorrectly or only partially make use of such techniques (Kebbell and Wagstaff, 1999; Kebbell et al., 1999; Clarke and Milne, 2001). This is frequently due to a lack of appropriate training or time constraints when conducting the interview. Therefore, in recent years researchers have focused on investigating simpler strategies to increase witnesses' accuracy that are easier to implement in practice. Dando et al. (2009), for instance, proposed a modified Cognitive Interview procedure based on the PEACE model, namely the Modified Peace Cognitive Interview Procedure (MPCI), in which mental reinstatement of context is replaced by a *sketch mental reinstatement of context* in which participants are asked to draw a sketch of the event to generate their own retrieval cues. In its shortened version, including a sketch free recall and a final free recall in lieu of a change of temporal order, this procedure proved to be effective and less time-consuming than the standard MPCI. Another valuable interviewing tool recently developed by Wagstaff and Wheatcroft (2010, Unpublished document; as cited in Wagstaff et al., 2011a,b) is the Liverpool Interview Protocol—a brief procedure for use in the field that combines the Focused Meditation, eye-closure, and context reinstatement elements.

An even simpler strategy is instructing witnesses to close their eyes during recall. When one has to focus on a task it is quite common for both children and adults to spontaneously close the eyes or look away in order to reduce interference from external sources and perform better (Doherty-Sneddon et al., 2002; Doherty-Sneddon and Phelps, 2005; Phelps et al., 2006; Markson and Paterson, 2009; Wais et al., 2010). In recent studies, instructing adults or children to close the eyes when recalling an event has been shown to increase the number of correct details reported, while at the same time decreasing the number of errors. Studies conducted with adults showed that eye-closure improves performance on mathematical and general-knowledge tests (Glenberg et al., 1998). Furthermore, eye-closure increases memory performance for visual details in a witnessed event (see Vredeveldt et al., 2012, 2013) and in some studies also for auditory details (see Perfect et al., 2008; Vredeveldt and Penrod, 2012). Studies conducted with children found that children instructed to avert their gaze (Phelps et al., 2006) or close their eyes (Mastroberardino et al., 2012; Natali et al., 2012) also perform better on arithmetic and verbal-reasoning tasks, and remember more correct information about witnessed events. Additionally, Natali et al. found that eye-closure increased children's memory accuracy for both visual and auditory details.

Based on their findings, Perfect et al. (2008) concluded that eye-closure has a *general* effect: it reduces cognitive load, resulting in benefits for recall of both visual and auditory details (see also Perfect et al., 2011, 2012). However, other findings that eye-closure predominantly benefits recall of visual details (Vredeveldt et al., 2012, 2013) point to a modality-specific effect: eye-closure reduces visual distractions in the environment, which specifically enhances performance on tasks that are visual in nature, such as recall of visual details. Vredeveldt et al. (2011) conducted a direct test of the general and modality-specific accounts of the eye-closure effect, respectively, by varying the nature of distractions during the interview. They found evidence for both general and modality-specific accounts. Thus, memory performance was better when distraction during the interview was minimal (most likely due to a reduction in general cognitive load). In addition, recall of visual material was most disrupted by exposure to visual distractions, whereas recall of auditory material was most disrupted by exposure to auditory distractions (i.e., a modality-specific interference effect). Finally, they found no significant difference between participants who closed their eyes and participants who looked at a blank screen during the interview, suggesting that reducing visual distractions in the environment is as effective as eye-closure.

The study conducted by Vredeveldt et al. (2011) suggests that, for adults, eye-closure reduces general cognitive load as well as modality-specific interference. However, it is not clear whether eye-closure has the same effects on children's performance. For example, two recent studies on the role of repeated recall and delay in the eye-closure effect, one conducted with adults (Vredeveldt et al., 2013) and one conducted with children (Natali et al., 2012), came to slightly different conclusions. In both studies, eye-closure during an interview taking place approximately 1 week after the witnessed event significantly improved recall performance. However, Natali et al. also found that children benefited from eye-closure during an interview taking place immediately after the event, whereas Vredeveldt et al. did not observe such benefits for adult participants. Thus, it is possible that eye-closure differentially affects memory in children and adults. A possible explanation for any differences between children and adults may relate to developmental differences. It is possible that the task of recalling information from a video seen immediately prior to the interview was not too cognitively demanding for adults. This could explain why eye-closure did not have an effect, since eye-closure is generally found to be most beneficial for cognitive tasks that are at least moderately difficult (cf. Glenberg et al., 1998). For children, on the other hand, even recall immediately after viewing an event may be a relatively difficult task, due to age differences in cognitive control capacity of attention shifting (see Enns, 1990, for a review). Thus, eye-closure may have helped children to allocate their attentional resources more effectively, by allowing them to disengage from irrelevant information in the environment and focusing their attention on the recall task.

In this study, we aim to investigate the relative influences of general and modality-specific components in the eye-closure effect in children. Based on previous research with adults (Vredeveldt et al., 2011), we hypothesized that (a) children exposed to minimal distraction during the memory task would provide more correct responses and fewer incorrect responses than children exposed to visual or auditory distractions, and (b) recall of visual material would be most disrupted by visual distractions, whereas recall of auditory material would be most disrupted by auditory distractions. However, due to differences related to development of cognitive control of voluntary attention (i.e., attention shifting, see Enns, 1990), we could not be certain that the same pattern would emerge in children.

## Methods

### Participants

One hundred and twenty children (60 female) aged between 8 and 11 years (*M* = 8.99; *SD* = 0.87) voluntarily participated in this study. Children were recruited from schools in Rome and had no familiarity with spoken or written Hebrew. This study was approved by the ethical committee of the Sapienza University of Rome and parents and teachers gave their informed consent before participation.

### Materials

All experimental materials were in Italian. A 5-min clip created for this experiment was used as study material (see Supplementary Material for a detailed description). The clip shows a series of events taking place in a private residence: a girl making a phone call, a parcel being delivered, the delivery man stealing a 50 Euros bill from a wallet, and a group of friends meeting up for a chat. In order to provide some memorable data to our participants, six clearly discriminable people appeared in the video, different scenes took place in clearly identifiable rooms in an apartment (i.e., kitchen, living room etc.), and names of the actors were clearly spoken.

### Design and procedure

This study employed a 4 (Interview Condition: blank screen, eyes closed, visual distraction, auditory distraction) × 2 (Modality of Encoded Information: visual, auditory) mixed design. All participants were tested individually in a small room during school hours. The experimenter welcomed the children and told them that they were going to see a short movie and that they had to answer some questions about it later. The clip was then presented on a 14.5' television screen. Following this, participants were randomly assigned to one of the four interview conditions and presented with an 18-item open-ended questionnaire (9 questions on visual and 9 questions on auditory details, see Supplementary Material). They were instructed to respond according to what they remembered and to avoid guessing by saying “don't know.” Participants in the blank screen condition (control group), were instructed to look at the blank screen throughout the interview, while participants in the eye closure condition were asked to keep their eyes closed. The visual- and auditory-distraction stimuli were identical to those used by Vredeveldt et al. (2011) with adult participants. Children in the visual distraction condition were instructed to look at the screen where Hebrew words (in Hebrew script) were presented in random locations (one per second), while participants in the auditory distraction condition looked at the blank screen while they heard Hebrew words being spoken (one per second). If, at any point during the interview, participants failed to follow the instruction (e.g., they looked away from the screen or opened their eyes) the interviewer reminded them what they were instructed to do at the beginning of the questioning phase. All children completed the experiment and after the questioning phase were fully debriefed and thanked for their participation.

## Results

A preliminary analysis showed no significant influence of age on participants' performance. The means and standard deviations for correct responses, incorrect responses, confabulated responses, and “don't know” (DK) responses are shown in Table 1. For correct and incorrect responses, we conducted 4 (Interview Condition: blank screen, eyes closed, visual distraction, auditory distraction) × 2 (Modality of Encoded Information: visual, auditory) mixed analyses of variance (ANOVA) with repeated measures on the second factor. For confabulated and DK responses, we conducted Kruskal-Wallis tests, because the data were positively skewed and leptokurtic, and transformations did not correct this.

**Table 1:** **Mean proportions and standard deviations (in parentheses) of correct, incorrect, confabulated, and “don't know” responses to questions about visual and auditory details in the four interview conditions**.

	**Interview condition**				
	**Blank screen**	**Eyes closed**	**Visual distraction**	**Auditory distraction**	**Total**
**VISUAL DETAILS**					
Correct	0.79 (0.12)	0.82 (0.12)	0.61 (0.13)	0.56 (0.16)	0.70 (0.17)
Incorrect	0.13 (0.11)	0.08 (0.09)	0.26 (0.13)	0.26 (0.16)	0.18 (0.15)
Confabulated	0.02 (0.06)	0.01 (0.03)	0.03 (0.05)	0.03 (0.08)	0.02 (0.06)
“Don't know”	0.06 (0.09)	0.08 (0.09)	0.10 (0.10)	0.14 (0.12)	0.09 (0.10)
**AUDITORY DETAILS**					
Correct	0.76 (0.16)	0.73 (0.16)	0.75 (0.17)	0.76 (0.16)	0.75 (0.16)
Incorrect	0.12 (0.13)	0.10 (0.16)	0.12 (0.11)	0.11 (0.11)	0.11 (0.13)
Confabulated	0.03 (0.06)	0.03 (0.05)	0.03 (0.05)	0.03 (0.05)	0.03 (0.06)
“Don't know”	0.07 (0.10)	0.12 (0.11)	0.09 (0.10)	0.08 (0.07)	0.09 (0.10)

### Correct responses

An ANOVA on proportion correct revealed a significant effect of modality of encoded information, *F*_(1, 116)_ = 9.92, *p* = 0.002, η^2^ = 0.06. This likely reflected that questions about visual details were somewhat more difficult (with an average of 70% correct) than questions about auditory details (75% correct). There was also a significant effect of interview condition, *F*_(3, 116)_ = 8.39, *p* < 0.001, η^2^ = 0.18; children performed better in the blank-screen and eyes-closed conditions than in the visual-distraction and auditory-distraction conditions (see Table 1). Finally, there was a significant interaction between modality and condition, *F*_(3, 116)_ = 14.54, *p* < 0.001, η^2^ = 0.26. Simple effects analyses showed that interview condition had a significant impact on correct responses about visual details of the witnessed event, *F*_(3, 116)_ = 26.95, *p* < 0.001, η^2^ = 0.41, but did not significantly affect correct responses about auditory details (*F* < 1). Pairwise comparisons for visual details (Bonferroni-corrected α = 0.008) confirmed that the differences between the blank-screen and eyes-closed conditions (*p* = 0.34) and between the visual- and auditory-distraction conditions (*p* = 0.21) were not significant, whereas all other differences between conditions were significant (all *ps* < 0.001). In sum, children in the blank-screen and eyes-closed conditions provided more correct responses about visual details than children in the visual- and auditory-distraction conditions. Moreover, eye-closure had large effects on correct recall of visual details, compared to both the visual-distraction (*d* = 1.67) and the auditory-distraction (*d* = 1.81) condition.

### Incorrect responses

Prior to analysis, the incorrect-response data were square-root transformed to reduce positive skew and leptokurtosis. The ANOVA revealed significant effects of interview condition, *F*_(3, 116)_ = 7.20, *p* < 0.001, η^2^ = 0.16, modality of encoded information, *F*_(1, 116)_ = 26.31, *p* < 0.001, η^2^ = 0.16, and a significant interaction between the two, *F*_(3, 116)_ = 7.34, *p* < 0.001, η^2^ = 0.13 (see Table 1). Simple effects analyses revealed that interview condition had a significant impact on incorrect responses about visual details, *F*_(3, 116)_ = 18.02, *p* < 0.001, η^2^ = 0.32, but not on incorrect responses about auditory details (*F* < 1). Pairwise comparisons for visual details (Bonferroni-corrected α = 0.008) confirmed that the differences between the blank-screen and eyes-closed conditions (*p* = 0.06) and between the visual- and auditory-distraction conditions (*p* = 0.62) were not significant, whereas all other differences between conditions were significant (all *p*s < 0.001). In sum, children in the blank-screen and eyes-closed condition gave fewer incorrect responses about visual details than children in the visual- and auditory-distraction conditions. Eye-closure had large effects on incorrect recall of visual details, compared to both the visual-distraction (*d* = −1.62) and the auditory-distraction (*d* = −1.36) condition.

### Confabulations

Kruskal-Wallis tests revealed no significant effects of interview condition on the total number of confabulations [*H*_(3)_ = 0.70, *p* = 0.87], confabulations about visual details [*H*_(3)_ = 1.09, *p* = 0.79], or confabulations about auditory details [*H*_(3)_ = 0.51, *p* = 0.93]. Table 1 shows that children provided very few confabulations overall. Thus, interpretation of these findings is difficult due to floor effects.

### “Don't know” responses

Kruskal-Wallis tests revealed significant effects of interview condition on the total number of DK responses [*H*_(3)_ = 9.18, *p* = 0.02] and on the number of DK responses to questions about visual details [*H*_(3)_ = 8.11, *p* = 0.04], but not on DK responses to questions about auditory details [*H*_(3)_ = 5.71, *p* = 0.12]. Mann-Whitney tests were used to follow up the significant effects. For both total and visual DK responses, only the contrast between the blank-screen condition and the auditory-distraction condition was significant at a Bonferroni-corrected α-level of.008 (total: *U* = 260, *p* = 0.003, η^2^ = 0.14; visual: *U* = 280, *p* = 0.007, η^2^ = 0.12). In sum, children in the blank-screen condition provided fewer DK responses about visual details than children in the auditory-distraction condition (*d* = −0.56).

## Discussion

We found that eye-closure (or looking at a blank screen) during recall substantially increased correct responses, and substantially decreased errors, for recall of visual information about the witnessed event, as compared to conditions in which children were exposed to visual and auditory distractions during the interview. Our findings with children only partly replicated Vredeveldt et al.'s (2011) findings with adults. Thus, we found a general effect of sensory distractions on recall of visual details, but we did not find a general effect on recall of auditory details. We also did not replicate their modality-specific effect (i.e., that recall of visual details was most impaired by visual distractions and that recall of auditory details was most impaired by auditory distractions).

In terms of visual distractions, our findings with children are in line with some findings with adults, but not others. First, Perfect et al. (2012) manipulated visual distractions directly, and found that increased visual distractions led to fewer correct and more incorrect responses about both visual and auditory details in the event. We replicated this finding with regards to visual details, but did not find that visual distractions impaired recall of auditory details. Second, some adult studies manipulating eye-closure have found that eye-closure improved recall of both visual and auditory details (Perfect et al., 2008 Experiments 3–5; Vredeveldt and Penrod, 2012). However, other studies have found that eye-closure had selective benefits for recall of visual details only (Perfect et al., Experiment 2; Vredeveldt et al., 2012, Experiment 1; Vredeveldt et al., 2013). Similarly, in the present study, distractions in the interview environment only affected recall of visual details.

In terms of auditory distractions, our findings with children are partly in line with what Perfect et al. (2011) found for adults, namely that auditory distractions increased the number of errors for visual details. However, unlike Perfect et al. (2011), (a) auditory distractions in the present study also decreased the number of correct responses for visual details, and (b) auditory distractions did not impair recall of auditory details. The latter finding is in line with Vredeveldt et al. (2012, Experiment 2), who found that auditory distractions did not impair adults' recall of auditory details (although they also found that auditory distractions did not impair recall of visual details either, unlike the present study). In sum, our findings show that children perform better when they are interviewed in a silent environment. However, if it is not possible to conduct the interview in a silent environment, eye-closure during recall may help interviewees to overcome accuracy impairments caused by auditory distractions (Perfect et al., 2011). Future research should investigate whether this compensatory effect of eye-closure is also observed with children.

We found that distractions during the interview did not interfere with children's recall performance in a modality-specific way. Both auditory and visual distraction impaired participants' recall of visual details. It appears that closing their eyes or looking at a blank screen helped children to focus on the task of recalling visual information, while any type of external distraction had a disruptive effect. This might be explained in the light of a cognitive load hypothesis (Lavie and Tsal, 1994; Lavie, 2005; Lavie and Lin, 2009; Sweller et al., 2011), which suggests that people have a limited amount of cognitive resources they can devote to cognitive tasks. Therefore, performance on a cognitive task (such as attempting to retrieve information about a witnessed event) will be impaired by any concurrent tasks that require cognitive resources (such as monitoring the environment during the interview). Closing the eyes may be a way to reduce interference of external stimulation and reduce rememberers' cognitive load, both increasing the capability of the witness to focus on the memorial image and decreasing the burden of monitoring the environment for social cues (Bond and Titus, 1983). Given that children are particularly affected by the social and environmental components of an interview (e.g., characteristics of the interviewer, social cues and types of requests; see Krähenbühl and Blades, 2006; Quas et al., 2007), a reduction in cognitive load might explain the effect of eye-closure in increasing children's accuracy. In the present study, children's performance was affected by any form of distraction, probably because they experienced difficulties on focusing and sustaining attention over time (Ruff and Rothbart, 1996; Dowsett and Livesey, 2000; NICHD Early Child Care Research Network, 2005). Unlike adults, where a modality-specific effect was found, visual/auditory distraction had a general disruptive effect on children's performance.

An alternative account emerges from developmental studies on working memory and specifically on the “storage and processing” functions of the central executive (see Gathercole, 2000, for a review). According to this explanation, as the individual has to process incoming data (e.g., visual/ auditory distraction) and at the same time recall information, there will be a lower amount of activation available to support processing. Case et al. (1982) suggested that the total processing space available remains constant over development and it is the operational efficiency that is increased over time. Our results suggest that the latter was still inadequate in our sample of children, therefore any form of distraction interfered with performance.

Our findings also suggest that children's recall of visual details is more vulnerable to external distractions than their recall of auditory details. Why this is the case, is not clear. Perhaps, this effect is related to task demand. Although our questions about visual and auditory details were carefully designed, it is possible that our participants found it easier to respond to the latter, as illustrated by their ability to sustain consistently high memory accuracy for auditory details, even when faced with distractions during the interview. A second possible explanation may relate to the study material. In our experiment, auditory material was mostly presented as spoken by an actor within a social interaction. This may have produced a bimodal advantage (i.e., audio-visual), resulting in an enhancement of participants' performance as compared to visual material that was presented unimodally (see Mastroberardino et al., 2008 for a review).

In sum, our findings show that eye-closure is an ecologically valid and inexpensive way of helping children to recall the visual aspects of an event. We found that eye-closure resulted in sizeable benefits for children's recall of visual information. One of the most important (and unique) selling points of the eye-closure instruction is that it not only increases correct recall, but also decreases incorrect recall. Further, unlike many other interview protocols, it does not require any training or additional interview time, and can be easily implemented in forensic settings. Nevertheless, when questioning children one has to take into account that they do not report spontaneously most of the information they remember and that they have to be prompted using appropriate questioning (Goodman and Melinder, 2007; Melinder et al., 2010). Therefore, more research needs to be conducted into possible associations between eye-closure and other interview strategies to enhance children's memory performance.

### Conflict of interest statement

The authors declare that the research was conducted in the absence of any commercial or financial relationships that could be construed as a potential conflict of interest.
