# Supplementary material for: Eye-closure increases children's memory accuracy for visual material
Source: Front Psychol. 2014 Mar 24;5:241. doi: 10.3389/fpsyg.2014.00241 (PMC3970005; doi:10.3389/fpsyg.2014.00241)
Supplement: Supplementary file 1 [file Presentation1.PDF]

Appendix A

The clip starts showing a girl, Sara, sitting at a kitchen table making a phone call to a friend to arrange an appointment for dinner. Once the phone call is over, the doorbell rings and she leaves the room to go to open for a delivery man. The man has a parcel for her, and he asks the girl to sign a receipt. Since he has no pen she asks the man to follow her into the kitchen where she looks for one without finding it, she therefore asks the man to wait in the kitchen and goes to look for a pen and leaves the room. While she is away, the delivery man sees her purse on the table, takes out the wallet, opens it and steals 50 Euros. He then quickly puts the wallet under a tea cloth on the table, the girl comes back, signs the receipt and he goes away. After a while, two of Sara's friends, Tony and Julia, ring the door bell and she goes to open. They start chatting while walking to the living room where they sit on the sofa. After a little while another couple of friends, David and Marcia, arrive and they join the others in the living room. They briefly talk about a holiday David and Marcia spent in a European city and then they all leave the apartment. Once alone Sara goes back to the kitchen and finds out her money has been stolen. She then calls the delivery company and the clip ends.

Appendix B (questions about auditory details in italics)

At the beginning of the clip a girl named Sara makes a phone call. Can you describe her in detail?

Sara calls her friend Laura on the phone to tell her something. What does she tell her?

The doorbell rings and Sara goes to open. Can you describe which objects are in the hallway?

Sara opens the door to a delivery man. The man says he needs a signature, what does Sara reply?

The two walk into the kitchen and Sara walks out for a moment. What does the man do once he's alone?

Can you describe the delivery man in detail?

The doorbell rings and two of Sara's friends, Tony and Julia, walk in. Tony tells Sara something about Julia, what does he say?

Sara tells her friend to come and sit with her. Can you describe the room in which they sit down?

Once in the living room Sara's friends take off their jackets and sit down. Can you describe Tony in detail?

While they are seated together Sara asks them if they want to go to the cinema on Wednesday, what does Tony reply?

While they are speaking the doorbell rings. What does Sara say?

Sara opens the door and there are two other friends, David and Marcia. What does David say he brought?

They join the others in the living room and start a conversation. Sara asks Manuela how long the video lasts, what does she reply?

Once they have seen the video, they are all standing in front of the sofa. Can you describe David in detail?

The group discusses what they have seen in the video. Where does Tony say he wanted to go next year?

Sara walks her friends out and then goes into the kitchen to check what is inside the package.

Which objects are on the table?

While Sara is opening the package she realizes that her wallet is in a different place. What does she do?

Once Sara finds out that her wallet is empty who does she say she wants to call?
